# Inferior Vena Cava Elongation and Collapse During Congenital Scoliosis Surgery: A Case Report

**DOI:** 10.7759/cureus.79966

**Published:** 2025-03-03

**Authors:** Daniela Mistretta, Nicolás Valls, Jose Grass

**Affiliations:** 1 Department of Anesthesia, Hospital Dr. Luis Calvo Mackenna, Santiago, CHL; 2 Department of Anesthesia, National Cancer Institute, Santiago, CHL; 3 Department of Traumatology and Orthopedics, Hospital Dr. Luis Calvo Mackenna, Santiago, CHL

**Keywords:** congenital scoliosis, hemodynamic instability, inferior vena cava, intraoperative monitoring, vascular compromise

## Abstract

Correction of congenital scoliosis presents unique challenges due to anatomical distortions that may compromise vascular structures. Vascular complications during scoliosis correction are rare but potentially life-threatening. We report the case of a 13-year-old female individual with severe congenital lumbar scoliosis attributed to an L3 hemivertebra fused with L4. The patient underwent posterior spinal instrumentation using pedicle screws and an attempted convex rod derotation. During derotation, the patient developed profound hypotension and tachycardia, with immediate restoration of blood pressure and heart rate upon reversal. Owing to the recurrence of these hemodynamic disturbances, the surgical team opted for an in situ rod contoured technique, achieving partial deformity correction. Postoperative CT angiography revealed a significant reduction of approximately 60% in the inferior vena cava (IVC) lumen at the L3 level, although no thrombus was identified.

This case illustrates the significant hemodynamic challenges that can arise from altered vascular anatomy in severe congenital scoliosis. It emphasizes the need for heightened awareness and careful preparation to manage vascular complications that may occur during complex spinal procedures. Comprehensive preoperative vascular imaging, combined with meticulous intraoperative monitoring, is essential for the early identification and management of risks such as IVC compression. Ultimately, the ability to promptly detect hemodynamic instability and adapt the surgical strategy is crucial in preventing potentially catastrophic outcomes during spinal deformity correction.

## Introduction

Congenital scoliosis is frequently associated with complex anatomical distortions that pose significant challenges during surgical intervention. These distortions can complicate corrective maneuvers, such as rod derotation or vertebral derotation, by inducing translation of the soft prevertebral structures, including major blood vessels [1,2]. In particular, elongation and compression of vascular structures, notably the inferior vena cava (IVC), have been documented in patients with severe spinal deformities. We report a rare case of intraoperative IVC elongation and collapse during posterior spinal instrumentation in a patient with congenital scoliosis, emphasizing the critical importance of meticulous preoperative planning and vigilant intraoperative monitoring.

## Case presentation

A 13-year-old female individual, born prematurely (birth weight: 1.6 kg), presented with progressive lumbar scoliosis diagnosed at age eight. She reported no other comorbidities, prior surgeries, or significant familial history of scoliosis or vascular abnormalities. Physical examination revealed a severe right-sided lumbar curve.

**Figure 1:**
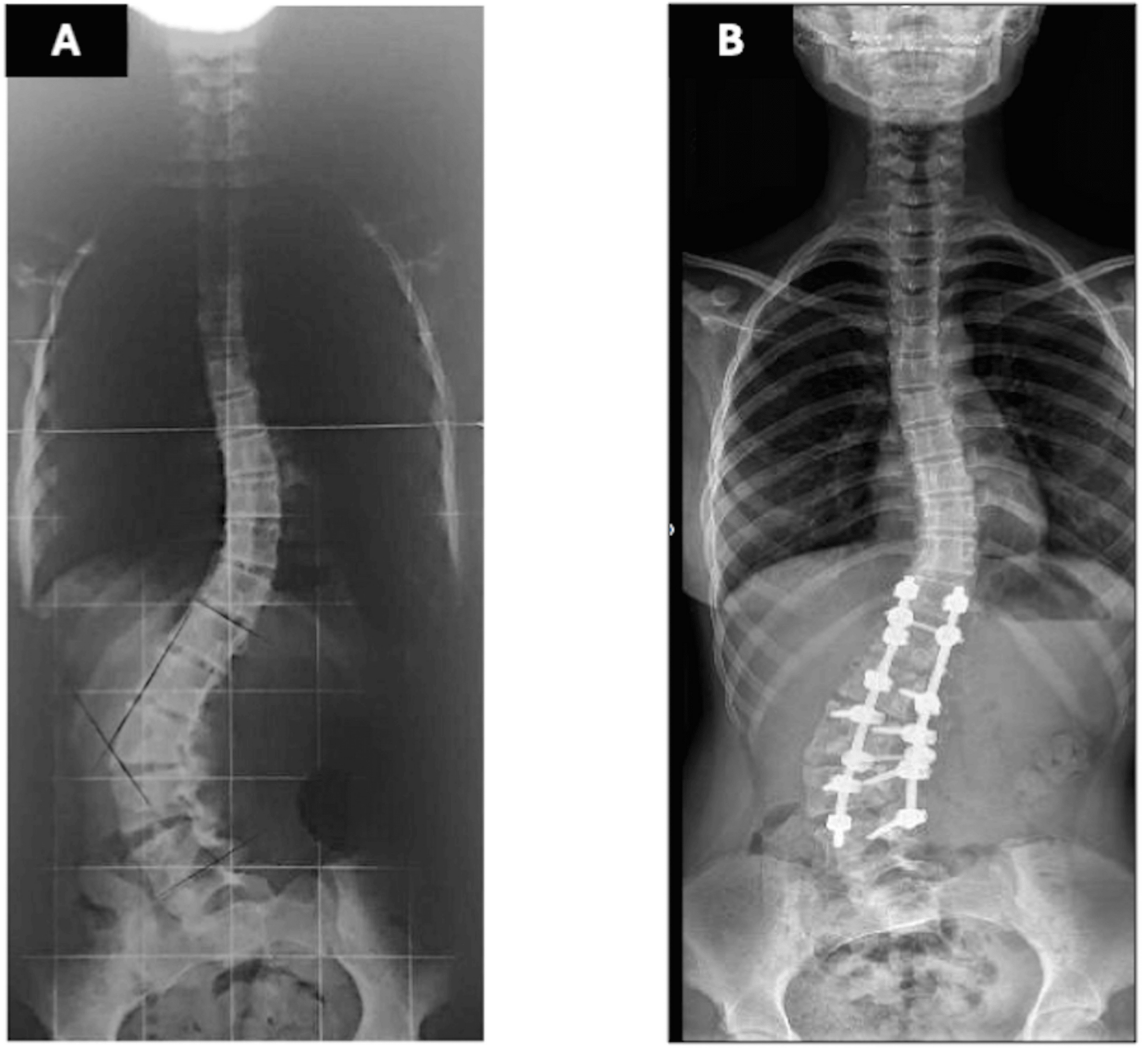
Anteroposterior spinal radiographs. (A) Preoperative radiograph: Severe scoliotic curve measuring 67°, presence of six lumbar vertebrae, and an L3 hemivertebra fused to L4. (B) Postoperative radiograph: Reduction of the scoliotic curve to 37° following a modification in the surgical plan.

Radiographs demonstrated a 67° lumbar scoliosis due to an L3 hemivertebra fused ventrally and dorsally with L4. Previous imaging showed progression from 22° at age eight to 54° at age 11. Preoperative laboratory tests were within normal limits, including hemoglobin of 13.6 g/dL (reference range: 12.0-14.0 g/dL) and hematocrit of 40% (reference range: 36-43%). Preoperative traction radiographs demonstrated partial curve correction to 37° (Figure 1a). A computed tomography (CT) evaluation confirmed the previously described characteristics, including a 67° lumbar scoliosis caused by a ventral and dorsal fusion of L3 and L4 vertebrae. Additionally, the preoperative CT scan revealed that the IVC and aorta were located within the concavity of the spinal curve, a finding commonly observed in scoliosis cases. No other abnormalities were detected in other organs.

It was proposed to the family to perform hemivertebra resection through an anterior and posterior approach. The family rejected the suggestion and agreed with the correction made through posterior spinal instrumentation with screws and vertebral derotation, although it was explained that the correction would be considerably less significant.

The anesthetic plan included total intravenous anesthesia with propofol and remifentanil. Monitoring included the American Society of Anesthesiologists standard [3], as well as invasive arterial pressure measurement, somatosensory and motor evoked potentials, and SedLine® (Irvine, CA) electroencephalography.

The patient was positioned prone on an Allen table. Bilateral pedicle screws were placed from T12 to L5, preserving the hemivertebra and L6. (L6 in orthopedic terminology denotes the lumbarization of the first sacral vertebra. This condition implies that the vertebra functions in a manner akin to a lumbar vertebra, rather than being an additional lumbar vertebra in the strict anatomical sense.) A 5.5-mm rod was inserted on the convex side, and vertebral derotation was attempted to convert the scoliotic curve into a lordotic alignment. During this maneuver, the patient developed profound hypotension (Mean Arterial Pressure < 50 mmHg) and tachycardia (heart rate > 140 bpm). Despite volume resuscitation and vasopressor administration (ephedrine and phenylephrine), hemodynamic stability was not maintained; however, approximately three minutes after reversing the derotation, blood pressure and heart rate recovered immediately. A subsequent vertebral derotation attempt made ten minutes later resulted in the recurrence of the same hemodynamic disturbance, which again resolved upon reversing the maneuver. Consequently, the surgical team opted to perform the vertebral derotation gradually, continuously monitoring hemodynamic parameters and reversing the maneuver incrementally at the first sign of instability until complete recovery was achieved. Because the correction achieved by derotation was deemed insufficient, an in-situ rod-bending technique was subsequently applied to obtain partial deformity correction (Figure 1b). Somatosensory and motor evoked potentials remained unaltered throughout the procedure. The surgery was completed without further complications, with an estimated blood loss of 500 mL and the administration of 2000 mL of Lactated Ringer’s solution.

**Figure 2:**
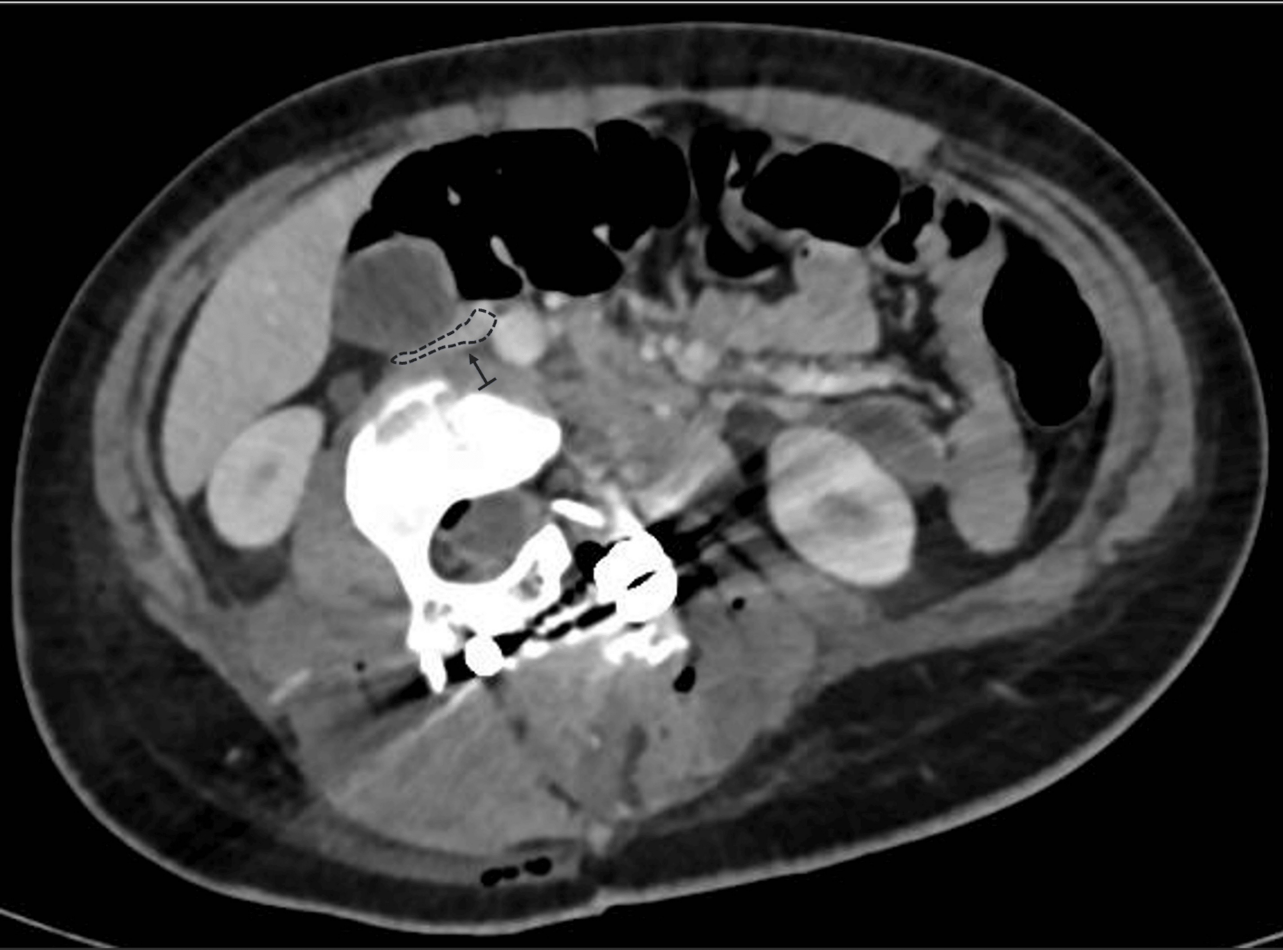
CT angiography; extrinsic compression of the inferior vena cava. The infrarenal inferior vena cava (indicated by the arrow and the dashed line) shows extrinsic compression. This compression reduces the venous lumen by approximately 60%.

The patient remained hemodynamically stable postoperatively but exhibited persistent sinus tachycardia. Laboratory tests revealed a drop in hemoglobin to 9.2 g/dL (reference range: 12.0-14.0 g/dL) and an elevation in lactate to 11.6 (reference range: 6.3-18.9 mg/dL) mg/dL. She received a transfusion of 460 mL of packed red blood cells, which improved her hemoglobin to 12 g/dL. Postoperative CT angiography demonstrated substantial compression and displacement of the IVC, resulting in a 60% reduction in its lumen diameter at the L3 level (Figure 2). No evidence of thrombus or venous return obstruction was found. Furthermore, cardiology evaluation and echocardiography were performed to rule out other differential diagnoses that might explain the intraoperative hemodynamic changes, thereby excluding cardiac abnormalities. Furthermore, it was determined not to administer anticoagulation to the patient, despite the IVC compression, based on the absence of venous stasis or thrombotic findings on imaging. This conservative approach aligns with previous recommendations suggesting targeted intervention only when hemodynamic or thrombotic complications are evident [2,4].

## Discussion

The present case underscores the complexity of managing hemodynamic instability caused by vascular elongation and compression during corrective surgery for severe congenital scoliosis. Although vascular complications in spinal deformity surgery are rare, they can be catastrophic if not promptly recognized and managed.

Regarding surgical technique, for patients younger than 10 years without secondary structural curves, the standard approach involves hemivertebra resection followed by fusion of the two adjacent vertebrae. If necessary, one or two additional segments may be incorporated into the fusion, contingent upon the curve’s length. In patients older than 10 years with more severe deformities and, in certain instances, secondary structural curves, hemivertebra resection is combined with an extended instrumented fusion to provide stability for the more extensive curves [5]. Consequently, brace treatment is not considered the preferred option in congenital scoliosis [6].

Initially, an anterior-posterior hemivertebra resection was proposed to the family. However, after discussing the associated risks, the family declined this option. An alternative approach aimed at achieving sub-optimal correction using pedicle screws and the convex rod rotation technique was then advised. This method was discussed and agreed upon by both the parents and the patient. Nevertheless, severe hemodynamic instability occurred during both attempts at this maneuver, it was deemed necessary to modify the primary correction technique to an in situ rod-contouring technique. This technique is one of the corrective maneuvers described for severe and rigid scoliosis and is typically employed in conjunction with other techniques [7].

The literature contains few reports of severe hypotension and shock during the derotation corrective maneuver. In most cases, neurogenic shock resulting from spinal cord injury is described. This occurs when the disruption of descending sympathetic pathways leads to unopposed vagal tone in vascular smooth muscle, causing decreased systemic vascular resistance, vasodilation, and relative bradycardia [8,9]. However, this phenomenon is unlikely to explain the hypotensive event in our patient, as somatosensory and motor evoked potentials remained unaltered throughout the entire procedure.

In severe scoliosis, the anatomical positions of the great vessels are markedly altered, with the IVC and aorta frequently residing within the concavity of the spinal curve. This displacement effectively shortens their vascular length relative to a neutral alignment. Thus, during surgical maneuvers that involve rotation or lengthening of the spinal column, the anterior structures are exposed to significant tension and compression forces. In our case, where the hemivertebra was fused with adjacent vertebrae, forming a substantial mass similar to a vertebral block, it is plausible that derotation, by increasing lumbar lordosis and facilitating the anterior translation of the apical block vertebrae, reduced the distance between the spine and the anterior abdominal wall. Moreover, this derotation may have contributed to spinal elongation and stretching of the anterior soft tissues. These mechanisms are supported by postoperative CT angiography, which demonstrated that the correction maneuver increased the anterior spinal length, exerting significant traction on the IVC and reducing its lumen diameter by approximately 50% at the L3 level. Similar findings have been reported in studies examining vascular shifts during scoliosis correction, indicating that the position and diameter of the IVC and aorta are highly dependent on the severity and rigidity of the deformity [1,4].

Considering the aforementioned factors, the severe hypotension and tachycardia observed intraoperatively were attributed to venous return obstruction caused by the elongation and compression of the IVC. While arterial blood flow generally remains unaffected due to the higher pressures within arterial vessels, venous structures are more susceptible to collapse under external forces. The disparity in circulatory dynamics resulted in a decrease in cardiac preload and output, which was clear during the derotation maneuver in this case [4,10]. This phenomenon aligns with prior observations in vascular surgery and scoliosis correction, where elongation or compression of the IVC has been linked to hemodynamic instability. In a systematic review, Weiss & Goodall (2008) emphasized that vascular complications are more likely in rigid and severe scoliosis due to the anatomical constraints and abnormal vascular trajectories [11]. Similarly, Yang et al. (2020) highlighted that during scoliosis surgery, the elongation and strain of the IVC and aorta significantly increase the risk of vascular impingement and subsequent instability [4].

Accurate preoperative imaging is essential for identifying potential risks in severe and rigid scoliosis surgery. Advanced imaging techniques such as CT angiography can map the spatial relationship between the spine and adjacent vascular structures, providing critical insights into the risk of compression or elongation during correction [4,11]. In this case, postoperative imaging confirmed significant compression and displacement of the IVC into the concavity of the deformity. While the absence of thrombus or obstruction at rest was reassuring, this finding emphasized the dynamic nature of vascular compression during surgical manipulation. Dynamic imaging modalities, such as upright CT or MRI, could offer additional information by simulating positional changes and their impact on venous return [4].

The intraoperative events in this case emphasize the paramount importance of adaptability in surgical strategy when confronted with unforeseen hemodynamic challenges. Although vertebral derotation is an effective technique in reducing scoliosis, it must be performed with caution in severe cases to avoid excessive traction on anterior structures, which may further reduce the distance between the spine and the anterior abdominal wall. In this instance, the decision to abandon derotation in favor of in-situ rod-bending effectively mitigated the risk of additional vascular compromise. This approach aligns with recommendations from studies advocating staged or less aggressive correction techniques for rigid deformities, particularly when vertebral shortening procedures such as hemivertebra resection or total corpectomy are either not feasible or considered too risky [4,10,12].

Moreover, real-time intraoperative monitoring, including hemodynamic and neurophysiological parameters and effective team communication, proved invaluable in promptly detecting and addressing the vascular compromise. The postoperative management of this patient underscores the importance of a multidisciplinary approach in managing complications related to scoliosis surgery. Continuous hemodynamic monitoring, timely imaging, and collaborative consultations with both cardiology and vascular surgery teams ensured a comprehensive evaluation and effective management plan.

## Conclusions

In conclusion, our case highlights a rare but significant complication, intraoperative IVC elongation and collapse, which occurred during corrective surgery for congenital scoliosis. The altered vascular anatomy inherent to severe spinal deformities predisposes patients to hemodynamic instability during vertebral derotation maneuvers. Comprehensive preoperative imaging to delineate vascular trajectories, vigilant intraoperative hemodynamic and neurophysiological monitoring, and readiness to modify the surgical technique are essential for ensuring patient safety. Furthermore, a multidisciplinary approach, early recognition of complications, and effective communication in the operating room are critical to improving outcomes in complex spinal surgeries.
